# Clinical and microbiological effect of pulsed xenon ultraviolet disinfection to reduce multidrug-resistant organisms in the intensive care unit in a Japanese hospital: a before-after study

**DOI:** 10.1186/s12879-020-4805-6

**Published:** 2020-01-29

**Authors:** Keita Morikane, Shoko Suzuki, Jun Yoshioka, Jun Yakuwa, Masaki Nakane, Kenji Nemoto

**Affiliations:** 1 Division of Infection Control and Clinical Laboratory, Yamagata University Hospital, 2-2-2 Iida-Nishi, Yamagata, 990-9585 Japan; 2 Division of Nursing, Yamagata University Hospital, 2-2-2 Iida-Nishi, Yamagata, 990-9585 Japan; 3 Division of Clinical Engineering, Yamagata University Hospital, 2-2-2 Iida-Nishi, Yamagata, 990-9585 Japan; 4 Department of Clinical Engineering, Gunma Paz University, 1-7-1 Toiya-cho, Takasaki, 370-0006 Japan; 5 Advanced Critical Care Center, Yamagata University Hospital, 2-2-2 Iida-Nishi, Yamagata, 990-9585 Japan; 6 Director and Chairman, Yamagata University Hospital, 2-2-2 Iida-Nishi, Yamagata, 990-9585 Japan

**Keywords:** Ultraviolet disinfection device, Multidrug-resistant organism, *Acinetobacter baumannii*, Environmental disinfection

## Abstract

**Background:**

No-touch environmental disinfection using ultraviolet devices has been highlighted in the past several years to control the transmission of multidrug-resistant organisms (MDROs). However, its effectiveness in non-US healthcare settings is yet to be examined. This study aimed to evaluate the effectiveness of disinfection by portable pulsed xenon ultraviolet (PX-UV) devices in controlling transmission of MDROs in a non-US healthcare setting.

**Methods:**

All patients admitted in the intensive care unit in a 629-bed tertiary referral hospital in Japan from August 2016 to February 2019 were enrolled. During the study period, PX-UV disinfection was added to manual terminal cleaning after every patient transfer/discharge. For microbiological evaluation, surfaces were selected for sampling by contact plates before/after manual cleaning and after PX-UV. After overnight incubation, colonies on the plates were counted.

**Results:**

The incidence of newly acquired methicillin-resistant *Staphylococcus aureus* (MRSA) declined significantly (13.8 to 9.9 per 10,000 patient days, incidence rate ratio 0.71, *p* = 0.002), as well as that of newly acquired drug-resistant *Acinetobacter* (48.5 to 18.1, 0.37, *p* < 0.001). The percent reduction of the microbiological burden by manual cleaning was 81%, but a further 59% reduction was achieved by PX-UV.

**Conclusions:**

PX-UV is effective in further reducing the microbial burden and controlling MDROs in a non-US healthcare setting.

## Background

Infection remains a significant cause of morbidity and mortality in the healthcare setting, despite international initiatives in infection control and prevention. Pathogens such as *Clostridioides difficile*, vancomycin-resistant enterococci (VRE) and multidrug-resistant *Acinetobacter* are especially difficult to deal with. These pathogens can easily reside in the healthcare environment [1] and are difficult to remove or eradicate by conventional environmental cleaning, typically by manual wiping with disinfectants and cloths [2]. Ultraviolet light disinfection has recently been used as an adjunct to terminal cleaning, and many studies have shown its effectiveness in reducing environmental contamination [3–5] and healthcare-associated transmission of these pathogens [6–12]. However, most of these studies [3, 4, 6–12] have been performed in the United States, where the majority of rooms are private. To our knowledge, there are no published studies investigating the clinical effectiveness of this novel technology in healthcare settings outside the United States.

In Yamagata University Hospital, there has been sporadic identification of two-drug resistant *Acinetobacter baumannii* (2DRA), which is resistant to two classes of antimicrobial: carbapenem and quinolone. In order to halt the transmission of this pathogen, interventions such as enhanced terminal cleaning by hypochlorous acid and strict contact precaution were implemented. This was partially successful; however, it did not lead to the eradication of transmission. Therefore, we decided to implement further intervention using pulsed xenon ultraviolet light (PX-UV).

The objective of this study was to evaluate the effect of PX-UV on the transmission of healthcare-associated pathogens and on the environmental contamination within a Japanese healthcare setting.

## Methods

### Study settings and design

This study was conducted at Yamagata University Hospital, a 629-bed academic tertiary referral hospital in Yamagata, Japan. The Yamagata University School of Medicine Institutional Review Board approved this study with a waiver of informed consent.

### Implementation and operation of the disinfection device

A PX-UV device (Xenex Disinfection Services, San Antonio, TX, USA) was introduced in the study hospital in November 2017. It was decided that this device would be used in the intensive care unit (ICU), since most of the newly detected 2DRA infections occurred in the ICU patient population. After education to clinical engineering staff, the device was deployed in the ICU environment. The ICU has six rooms and beds. One room has independent walls and positive/negative air pressure. The other rooms are in an open space separated by curtains. Portable drapes, which protect UV and visible light from leaking outside the designated room, were used when operating PX-UV in the open space rooms. The device came into full operation by January 2018.

Terminal cleaning after every patient discharge or transfer to the ward was performed using cloths soaked with diluted sodium hypochlorite solution, which were then applied to every possible touchable surface in the room as well as portable and non-portable equipment. This type of cleaning was already implemented at the beginning of the baseline period and continued throughout the study period. Next, the room was covered by portable drapes, which were hung inside of the curtains of the room. After hanging the portable drapes, two 5-min disinfection cycles using PX-UV were run following the manufacturer’s recommendation. The machine was operated by clinical engineering staff, and sequentially placed on two opposite corners of the room. For each operation, the operator’s name, date and time, and duration (in seconds) of UV irradiation was recorded on the device’s cloud-based reporting system.

### Active surveillance of MDROs

Every patient admitted in the ICU was screened by nasal sampling for methicillin-resistant *Staphylococcus aureus* (MRSA) and 2DRA colonization on the day of the admission and every Tuesday and Thursday, until transfer or discharge from the unit. New acquisition of MRSA or 2DRA was defined as the identification of these pathogens from the screening specimen or clinical culture specimen on or after the third day of admission to the ICU, with at least one preceding negative screening result.

### Microbiological evaluation

Microbiological contamination of the environment was evaluated by using Replicate Organism Detection and Counting (Rodac) plates (P34 Tryptic Soy Agar with Lecithin and Tween 80, Hardy Diagnostics, Santa Maria, CA, USA). Ten frequently touched surfaces were selected for sampling: the bed rail (inside and outside near patient’s head, near patient’s feet), touch panel of cardiopulmonary monitor, ventilator control panel, intravenous fluid pump control panel, glove hanger, workstation keyboard, workstation cart handle and water basin. Samples were collected by gently stamping Rodac plates onto the site for 10 s. The roll plate method was used for nonflat surfaces such as bedrails. Sampling occasions included (1) immediately after patient discharge, before terminal cleaning (2) immediately after terminal cleaning (3) after PX-UV disinfection. Plates were then cultured at 37 degrees Celsius for 18 h. Colony-forming units (CFU) were counted and reported as CFU per 25cm [2].

### Statistical analysis

Poisson regression model analysis was used to estimate the incidence of MRSA and 2DRA infection, which were expressed as the number of acquisitions per 10,000 patient days. For the statistical analysis, we assumed that the pre-intervention period was the baseline, which means the event (acquisition of MRSA or 2DRA) occurs in a probability calculated by using the data in the pre-intervention period. To analyze the CFU data a Shapiro-Wilk test was conducted to determine the skewness of the CFU data. The results suggested the CFU data were skewed (*p* < 0.001), indicating the need for a nonparametric test. The Dunn’s Test of multiple comparisons using a rank sum was used to assess the difference between environmental sampling timepoints. This test is a nonparametric multiple comparison test, that uses a Wilcoxon Rank Sum that allows for more than a two-group comparison. The Dunn’s Test used a Bonferroni correction to adjust for multiple comparisons. The statistical analyses were conducted using the Poisson and Dunn’s Test package in STATA 15.1 (College Station, TX, USA).

## Results

### Change in the incidences of MDROs

The baseline incidence of MRSA and 2DRA in the ICU were 13.8 and 48.5 per 10,000 patient days, respectively. The incidence dropped to 9.9 and 18.1, resulting in a decrease of 29 and 63%, respectively (Table 1). The reductions in both MRSA and 2DRA were statistically significant (*p* = 0.002 and *p* < 0.001, respectively) (Table 2). Notably, in the intervention period, new acquisition of 2DRA in the ICU was observed only in the first six months, with no new acquisition for a seven month period. This also led to the eradication of new acquisition of 2DRA throughout the hospital in August 2018.

**Table 1:** MRSA and 2DRA Infection, by locations

Aug 2016 – Jan 2018 (Baseline period)	ICU MRSA	HCU MRSA	All Other Units MRSA	ICU 2DRA	HCU 2DRA	All other Units 2DRA
Infections	4	5	44	14	13	17
Patient days	2852	6494	309,512	2852	6494	309,512
Infection incidence	13.84	7.64	1.23	48.5	20.2	0.55
Feb 2018 – Feb 2019 (Intervention period)	ICU MRSA	HCU MRSA	All Other Units MRSA	ICU 2DRA	HCU 2DRA	All other Units 2DRA
Infections	2	3	31	4	3	7
Patient days	2102	4554	218,907	2102	4554	218,907
Infection incidence	9.89	6.60	1.41	18.10	6.46	0.31
Percent Change	−28.5%	−13.6%	+ 14.6%	−62.6%	− 68.0%	−43.6%

NOTE. *ICU* intensive care unit, *HCU* high care unit, *MRSA* methicillin-resistant *Staphylococcus aureus*, *2DRA* two-drug resistant *Acinetobacter baumannii*

**Table 2:** Poisson models

	ICU MRSA	HCU MRSA	All Other Units MRSA	ICU 2DRA	HCU 2DRA	All other Units 2DRA
Incidence Rate Ratio	0.71	0.86	1.14	0.37	0.31	0.56
95% CI	0.57–0.88	0.65–1.12	0.62–2.12	0.32–0.43	0.25–0.40	0.18–1.80
p-value	0.002	0.283	0.663	< 0.001	< 0.001	0.338

NOTE. *ICU* intensive care unit, *HCU* high care unit, *MRSA* methicillin-resistant *Staphylococcus aureus*, *2DRA* two-drug resistant *Acinetobacter baumannii*

### Microbiological effect

Environmental sampling was performed after 17 patient discharges. Some of the samplings were not performed due to time constraints by waiting patients or unique features of certain rooms. In total, 128 sites were sampled. The total colony count was: 3336 (before manual cleaning), 669 (after manual cleaning, before PX-UV) and 280 (after PX-UV). Compared to pre-cleaning, a statistically significant decrease in colony count was observed after manual cleaning (*p* < 0.0001, Wilcoxson rank sum test) (Table 3). Also, compared to post-manual cleaning, a statistically significant decrease in colony count was observed after PX-UV disinfection (*p* = 0.0213) (Table 3).

**Table 3:** Reduction in colony-forming units (CFU) from the environment

	Before Cleaning (A)	After Cleaning (B)	After PX-UV (C)
Total CFU	3336	679	280
Median (IQR)	10 (2–30)	0 (0–2)	0 (0–1)
A vs B* (*p*-value)	**–**	79.6% (p < 0.0001)	–
A vs C* (*p*-value)	**–**	–	91.6% (*p* < 0.0001)
B vs C* (*p*-value)	**–**	**–**	58.7% (p = 0.0213)

NOTE: *IQR* Inter-quartile range, *PX-UV* pulsed xenon ultraviolet

*Bonferroni correction used to adjust for multiple comparisons

## Discussion

There are a number of studies which have demonstrated the effect of ultraviolet light disinfection in reducing environmental microbiological contamination [3–5] and healthcare-acquired infections by MRSA [6, 7], VRE [7, 8] and *Clostridioides difficile* [7–12]. However, all but one study [5] was performed in the United States, where most of the patient rooms are private. Furthermore, that study [5] did not evaluate clinical outcomes.

Our study is the first hospital-acquired infection outcome study evaluating the clinical effectiveness of the PX-UV device in a non-US healthcare setting with an open-style ICU. In this setting, patient beds were separated by privacy curtains, not by walls. PX-UV emits intense visible light and creates a sound while disinfecting. The light and sound can be seen and heard outside the privacy curtains. This was initially not well tolerated by healthcare staff, some of whom raised concerns about sensitivity to the light and sound. We also experienced faults from the pulse oximeter when PX-UV was used adjacent to patient beds. To overcome these challenges, we provided goggles and earplugs to healthcare staff and ordered blackout curtains from the PX-UV vendor that hung inside the privacy curtains during PX-UV operation. The blackout curtains worked well and eliminated the problems stated above.

Adding PX-UV disinfection to routine terminal cleaning after patient discharge increased the turn-around time of ICU beds by approximately 20 min. Manual cleaning by sodium hypochlorite solution took about 50 min, so the increase in time by adding PX-UV was not significant and was well accepted by ICU staff and physicians as a routine workflow.

The effectiveness of PX-UV is expected to be maximized when environmental contamination is a major factor in transmission of the specific pathogen. In this context, pathogens such as *Clostridioides difficile* and multidrug-resistant *Acinetobacter* are more likely to be controlled. The effectiveness of PX-UV in controlling transmission of *Clostridioides difficile* is well studied and demonstrated [7–12], but that of *Acinetobacter* has not been well investigated.

Sporadic transmission of 2DRA has been observed in our ICU for the last five years. In Japan, antimicrobial resistance of *Acinetobacter* is not as serious. According to the Japanese national microbiological surveillance report, 97, 97 and 87% of *Acinetobacter* isolates were reported to be susceptible to meropenem, amikacin and levofloxacin, respectively [13]. Therefore, most of the newly identified 2DRA in our hospital seemed to be acquired by horizontal transmission. Until mid-2018, this situation has not been well controlled, despite our efforts for elevated compliance to hand hygiene, strict contact precaution of patients with this pathogen, terminal cleaning using bleach, and in some occasions, restriction of new admission to the ICU. However, by introducing the PX-UV, transmission of 2DRA in the ICU halted. As of June 2019, no new isolation of 2DRA from patients in the ICU has been observed for 11 months (August 2018 to June 2019). Generally, terminal cleaning by bleach is effective in preventing transmission of pathogens via environmental route, however, the result from the environmental sampling in this study, approximately 80% reduction in CFU by manual cleaning, indicated that cleaning by bleach is partially effective in reducing the bioburden in the patient care area. The halt of transmission of 2DRA in our ICU after introducing PX-UV suggests its adjunctive effect.

The effectiveness of ultraviolet light disinfection is maximized when performed after every patient discharge from the targeted ward. In the only multicenter, randomized controlled study conducted by Anderson et al. [8], ultraviolet disinfection was performed in only isolation rooms occupied by known MDRO or *C. difficile* colonization or infection. They did not observe any statistically significant effect on the incidence of MRSA and multidrug-resistant *Acinetobacter*. The effect of the addition of PX-UV to terminal cleaning by bleach was not observed in the study by Anderson et al. [8]. The differences between our study result and the results from Andersen et al. could be due to differences in the healthcare setting (proportion of private rooms), in the way the device is implemented (disinfecting isolation rooms in multiple units vs every discharge/transfer on single unit), or in the intervention fidelity (proportion of eligible rooms that are disinfected). We operated PX-UV after every patient discharge or transfer from the ICU, regardless of their colonization status, and obtained a statistically significant reduction in the incidence of healthcare-associated transmission of MRSA and 2DRA. This difference may be because undetected carriers of MRSA or *Acinetobacter* could serve as a source of environmental contamination even under the operation of ultraviolet light disinfection in the targeted rooms.

No new isolation of 2DRA from patients in the non-ICU ward has been observed for 10 months (September 2018 to June 2019). We believe that PX-UV successfully decreased or eradicated the environmental *Acinetobacter* bioburden of not only the targeted ward (ICU), but also of the other wards, perhaps indirectly through the decrease in bioburden throughout the hospital and also of colonized/infected patients with 2DRA. Anderson et al. experienced a similar decrease in the transmission of MRSA and VRE throughout the hospital by using ultraviolet light disinfection in only isolation rooms after patients with targeted pathogen were discharged [8].

Our study has several limitations. First, the effect of PX-UV was evaluated using historical controls when we were not using this technology. We have only one ICU in our hospital, so we were not able to have a non-intervention arm in this study. Second, not all MRSA or 2DRA identified were necessarily acquired in the ICU by horizontal transmission. However, we screened all patients on the day of their admission into the ICU and if they were positive for MRSA and/or 2DRA we regarded it as prior acquisition and excluded them from the acquisition in the ICU. Therefore, we believe that most of the identified MRSA and 2DRA in this study were acquired by horizontal transmission. Third, the microbiological effect of PX-UV was evaluated by comparing the number of colonies from sampling the same frequently touched surface, but with different sites adjacent to each other. If there were significant differences in contamination between sites on the same surface, the result may not accurately reflect the effect of cleaning and PX-UV. We, however, believe that by sampling over 100 surfaces, we can minimize the effect by the heterogeneity of environmental contamination. Fourth, we did not measure the environmental contamination of MRSA or 2DRA by using selective media to sample the environment. For this reason, we are unable to demonstrate the direct relationship between environmental disinfection by PX-UV and the decrease/eradication of new isolation of MRSA or 2DRA. However, it is well known that contamination of the patient care area by the same pathogen that is isolated from the patient is common [14]. We have previously observed contamination by 2DRA on the keyboard in a patient care area where 2DRA was isolated from the patient (data not shown). Furthermore, MRSA, 2DRA and other common environmental flora are susceptible to bleach and PX-UV disinfection. Therefore, it is plausible that environmental contamination by MRSA and 2DRA was controlled by adding PX-UV as an adjunct to manual cleaning, which led to the decrease/eradication of the transmission of these pathogens.

## Conclusion

The addition of PX-UV to terminal cleaning successfully decreased the bioburden in the healthcare environment and led to the decrease of MRSA and drug-resistant *Acinetobacter* transmission in the ICU as well as in other wards of our hospital.

## Data Availability

The datasets generated and/or analysed during the current study are available in a GitHub repository, https://github.com/keitamorikane/BMCID.

## Abbreviations

2DRA: two-drug resistant *Acinetobacter baumannii*
CFU: colony-forming units
ICU: intensive care unit
MDROs: multidrug-resistant organisms
MRSA: methicillin-resistant *Staphylococcus aureus*
PX-UV: pulsed xenon ultraviolet
VRE: vancomycin-resistant enterococci
